# Depressive symptoms and frailty, effects of social distancing and isolation in older adults

**DOI:** 10.17533/udea.iee.v43n1e10

**Published:** 2025-04-28

**Authors:** Maria Helena Lenardt, Aline de Souza Falcão, Clovis Cechinel, João Alberto Martins Rodrigues, Susanne Elero Betiolli

**Affiliations:** 1 Nurse, Ph.D. Email: curitiba.helena@gmail.com. https://orcid.org/0000-0001-8309-4003 Universidade Federal do Paraná Brazil curitiba.helena@gmail.com; 2 Nurse, Master. Email: alinesousafalcao@hotmail.com. https://orcid.org/0000-0001-6060-041X Universidade Federal do Paraná Brazil alinesousafalcao@hotmail.com; 3 Physician, Ph.D. Email: cechinelc@hotmail.com. https://orcid.org/0000-0002-9981-3655 Universidade Federal do Paraná Brazil cechinelc@hotmail.com; 5 Nurse, Ph.D. Email: susanne.elero@yahoo.com.br. https://orcid.org/0000-0002-5708-3148 Universidade Federal do Paraná Brazil susanne.elero@yahoo.com.br; 6 . Universidade Federal do Paraná (UFPR), Curitiba, Paraná, Brazil. https://orcid.org/0000-0003-4469-4473 Universidade Federal do Paraná Universidade Federal do Paraná (UFPR) Curitiba Paraná Brazil

**Keywords:** coronavirus infections, social isolation, frail elderly, depression, primary health care., infecciones por coronavirus, aislamiento social, persona mayor frágil, depresión, atención primaria de salud., infecções por coronavírus, isolamento social, idoso fragilizado, depressão, atenção primária à saúde.

## Abstract

**Objective.:**

To analyze the effects of social distancing and isolation during the Covid-19 pandemic on depressive symptoms and frailty in older adults in Primary Health Care.

**Methods.:**

Prospective cohort study, using the following data collection instruments: Center for Epidemiological Studies depression scale, adherence to social distancing and isolation, and markers of the physical frailty phenotype. Descriptive statistical analysis, association and proportional hazards regression were performed.

**Results.:**

51.8% of the older adults progressed to pre-frailty, 14.1% had depressive symptoms, and a low degree of adherence to social distancing and isolation (69.4%). There was no association between distancing and isolation and depressive symptoms (*p*=0.748) and physical frailty (*p*=0.5). Single, separated, divorced or widowed people have 62% (HR=0.38; 95%CI 0.15-0.96) less risk of being classified as frail and 57% (HR=0.43; 95%CI 0.21- 0.9) less chance of presenting depressive symptoms compared to married people.

**Conclusion:**

the low degree of adherence to social distancing and isolation showed no association with depressive symptoms and physical frailty. Sociodemographic factors highlighted risks of frailty and depressive symptoms that require attention and an individualized gerontological care plan.

## Resumen

**Objetivo.:**

Analizar los efectos del distanciamiento social y del aislamiento social durante la pandemia de Covid-19 sobre los síntomas depresivos y la fragilidad en personas mayores en Atención Primaria de Salud.

**Métodos.:**

Estudio de cohorte prospectivo, en el que se utilizaron como instrumentos de recolección de datos: la escala de depresión del Center for Epidemiological Studies (CES-D), la escala de adherencia al distanciamiento y aislamiento social, y marcadores del fenotipo de fragilidad física. Se realizaron análisis estadísticos descriptivos, asociación y regresión de riesgos proporcionales.

**Resultados.:**

51.8% de las personas mayores evolucionaron a prefragilidad y 14.1% para síntomas depresivos, con bajo grado de adherencia al distanciamiento y aislamiento social (69.4%). No hubo asociación entre distanciamiento y aislamiento social y síntomas depresivos (*p*=0.748) y fragilidad física (*p*=0.5). Las personas solteras, separadas, divorciadas o viudas tienen un riesgo 62% (HR=0.38; IC95% 0.15-0.96) menor de ser clasificadas como frágiles y 57% (HR=0.43; IC95% 0.21-0.9) menor de presentar síntomas depresivos en comparación con las personas casadas.

**Conclusión.:**

El bajo grado de adherencia al distanciamiento y aislamiento social no mostró asociación con síntomas depresivos y fragilidad física. Los factores sociodemográficos que se relacionaron con riesgos de fragilidad y síntomas depresivos requieren atención y un plan de cuidado gerontológico individualizado.

## Resumo

**Objetivo.:**

Analisar os efeitos do distanciamento e isolamento social na pandemia Covid-19 nos sintomas depressivos e na fragilidade em pessoas idosas da Atenção Primária à Saúde.

**Métodos.:**

Estudo de coorte prospectivo, mediante os instrumentos de coleta de dados: escala de depressão Center for Epidemiological Studies (CES-D), adesão ao distanciamento e isolamento social e marcadores do fenótipo da fragilidade física. Foram realizadas análises estatísticas descritivas, associação e regressão de riscos proporcionais.

**Resultados.:**

51.8% das pessoas idosas evoluíram para pré-fragilidade e 14.1% para sintomas depressivos, e predominou baixo grau de adesão ao distanciamento e isolamento social (69.4%). Não houve associação entre distanciamento e isolamento social e os sintomas depressivos (*p*=0.748) e fragilidade física (*p*=0.5). Solteiros, separados, divorciados ou viúvos têm 62% (HR=0.38; IC95% 0.15-0.96) menos risco de serem classificados como frágeis e 57% (HR=0.43; IC95% 0.21-0.9) menos chance de apresentarem sintomas depressivos quando comparados aos casados.

**Conclusão.:**

O baixo grau de adesão ao distanciamento e isolamento não mostrou associação com sintomas depressivos e fragilidade física. Fatores sociodemográficos apontaram riscos de fragilidade e sintomas depressivos que necessitam atenção e plano de cuidado gerontológicos individualizados.

## Introduction

Preventive measures such as social distancing and isolation, respiratory etiquette and hand hygiene were encouraged as a way to contain the spread of Covid-19.^1^ Social distancing stands out among the measures adopted; it consists of “a conscious effort to reduce interactions between people in a larger community, in which individuals may be infected although not yet identified and therefore, not yet isolated”. Social isolation, in turn, is the “measure that refers to the separation of people infected with contagious diseases from uninfected people”.^2^ Both social isolation and social distancing were analyzed as preventive actions during the Covid-19 pandemic, a phenomenon that went beyond issues related to health measures, with biopsychosocial manifestations. Social isolation is a measure in which people are advised not to leave their homes in order to prevent the spread of the virus,^3^ while social distancing is a preventive measure in which people maintain a minimum distance between one another and avoid crowds.^4^

Although necessary, these restrictive measures have significant consequences on the lives of older adults. Restricting social and family interactions contributes to the risk of loneliness and predicts cognitive decline and decreased overall functionality.^5^ A longitudinal study conducted with data from the English Longitudinal Study of Aging (ELSA) assessed trends in frailty status associated with loneliness and social isolation over 14 years in a sample of 9,171 English people (mean age of 66.3 years), and presenting a high level of social isolation was associated with a higher frailty index score (p <0,0001).^6^

The relationship between social distancing and social isolation and the development of physical frailty is not fully understood. A study that evaluated the effects of social distancing and isolation on the frailty condition and physical activities of 168 community-dwelling older adults found no association between the variables.^7^ Physical frailty can be defined as “a clinical condition in which there is an increased vulnerability of an individual to the development of dependency and/or increased mortality when exposed to a stressor”.^8^ This condition is often assessed according to the phenotype proposed from the Cardiovascular Health Study (CHS), which consists of five biological markers: unintentional weight loss, self-reported fatigue/exhaustion, decreased handgrip strength, decreased physical activity, and reduced gait speed. The presence of three or more markers is classified as frail, one or two as pre-frail, and no marker as non-frail.^9^ Although widely recognized, there is no current established definition for depressive symptoms. They are often distinguished by depressive symptoms manifesting subtly with dysphoria and somatic symptoms, and are often associated with features of depression.^10^

A cross-sectional study conducted in Singapore with a sample of 721 older adults (≥60 years) investigated the association between the level of frailty and depressive symptoms. An increase in depressive symptom scores (p<0.001) was identified with the worsening of frailty.^11^ Social disconnection and decreased family interaction affect older adults, since most are not accustomed to digital technologies, which interferes with their social engagement. These circumstances together with social isolation may trigger the emergence/worsening of depressive symptoms.^12^

Studies on the possible consequences generated by social restrictions can contribute to society and especially to the population of older adults and encourage the adoption of alternative measures that help prevent and/or mitigate these consequences. Given the above, the aim of the present study was to analyze the effects of social distancing and social isolation during the Covid-19 pandemic on the frailty and depressive symptoms of older adults in Primary Health Care.

## Methods

This is a prospective cohort study developed from the formation of a cohort of participants who were identified without the variables of interest (depressive symptoms and physical frailty) in the pre-pandemic period. Subsequently, they were allocated into two cohorts according to the degree of exposure to social distancing and social isolation during the Covid-19 pandemic. The cohorts were monitored during the pandemic period and observed for the occurrence or not of the variables of interest. The study location was defined as a Basic Health Unit (UBS) in the southern region of Brazil (first wave of collection) and the households in the area covered by the same UBS (second wave of collection).

### Population, selection criteria and collection period and sample

Non-frail participants without depressive symptoms (n=147) were selected from the sample of 389 older adults to compose the first wave of the study, collected in 2019. The study cohorts were organized during the second wave of data collection, according to the degree of social distancing and social isolation (exposure) and observed for the occurrence of the variables of interest, depressive symptoms and/or physical frailty (outcome).

The target population consisted of older adults aged 60 years or older who were permanently registered at the UBS, had cognitive capacity to answer the questions or were accompanied by a caregiver, were not frail and free of the “depressive symptoms” marker in the first wave and had participated in both waves of data collection. The Mini-Mental State Examination (MMSE)^13^ was used for cognitive screening, and the level of education was considered for cutoff points.^14^

The exclusion criteria were: having a medical diagnosis of dementia, schizophrenia, Parkinson’s, being a wheelchair user or having had an amputation of the lower or upper limbs. The criteria for discontinuation were: death; moving address outside the coverage area of the UBS; being hospitalized during the data collection period; not being able to locate the registered address; and refusing to participate in the second wave. Of the 147 participants in the first wave of assessment, six died, 10 changed their address (outside the area covered by the UBS), one was hospitalized, 15 were deregistered from the UBS, 29 refused to participate, and one was not located at home. Thus, the sample for the second wave of evaluation consisted of 85 older adults.

The second wave of evaluation was during the pandemic period, between July and August 2021. The diagnosis of Covid-19, time of isolation in case of suspected Covid-19, and the Scale of Degree of Adherence to Social Distancing and Social Isolation were the variables collected. The total time between the first and second waves of data of collection was two years and seven months.

### Study variables and data collection

The dependent variables were the presence of depressive symptoms and the condition of physical frailty. The independent variables were sociodemographic, clinical, and degree of social isolation and social distancing. The structured questionnaire included sociodemographic identification questions (sex, age, race, marital status, education, and financial situation) and clinical questions (health status, use of medications, polypharmacy, hospitalizations, and report of falls in the previous year).

**Figure 1:**
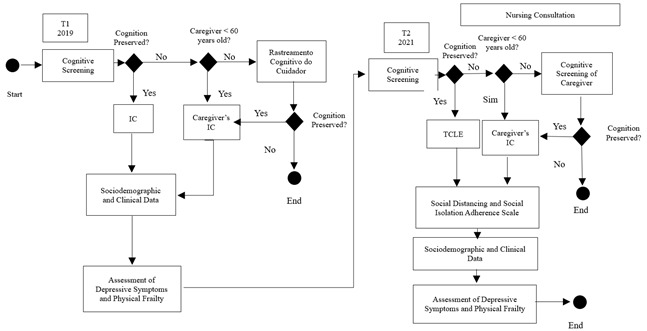
Flowchart of the study data collection steps.

To identify the condition of physical frailty, the markers of the frailty phenotype by Fried et al.(2001) were assessed: slow walking speed, poor handgrip strength, unintentional weight loss, decreased level of physical activity, and self-reported fatigue/exhaustion.^9^ Depressive symptoms were evaluated by applying the Center for Epidemiological Studies Depression Scale (CES-D). This screening instrument has 20 items that address mood, somatic symptoms, interactions with others, and motor functioning. Responses are determined by the Likert scale: never or rarely (0), a few times (1), almost always (2), always (3). The score ranges from 0 to 60 points. A score >11 indicates the presence of depressive symptoms^15^. To assess the degree of adherence to social distancing and social isolation, a Likert-type scale was developed by the authors of this study. After explaining the concepts of social distancing and social isolation, study participants were asked to recall the period from March to December 2020, the period before the application of vaccines for immunization against Covid-19. The following questions were used: Did you practice social distancing? Did you practice social isolation? Did you practice this distancing and/or social isolation without leaving home? For each question, the answer is categorized as: (1) never (<10 days), (2) rarely (10-14 days), (3) sometimes (15-30 days), (4) almost always (31-90 days), (5) always (>90 days). Scores four (4) and five (5) are considered to be a high degree of adherence, and the others represent a low degree of adherence. Older adults were categorized into two cohorts based on the degree of adherence to social distancing and social isolation. Figure 1 shows the flowchart of the study data collection steps.

### Data analysis and processing

Descriptive statistical analysis techniques were used to assess changes in terms of sociodemographic and clinical characteristics, condition and markers of frailty, depressive symptoms, and degree of adherence to social distancing and social isolation. Individuals who did not provide complete information, present in the first and second waves, were excluded. The cohorts of the first and second waves were compared with the group of losses for the treatment of losses to follow-up and assessment of any statistical difference between proportions. Then, the chi-square test for n ≥ 5 and the Fisher's exact test for n < 5 were applied to test the associations between depressive symptoms and frailty with the degree of adherence to social distancing and social isolation.

The time variable was constructed by subtracting the date of collection of the information of the second wave from the date of the first wave. Semi-parametric Cox proportional hazards models were conducted to obtain adjusted estimates of hazard ratios. Sociodemographic variables not indicated by the adjustment model due to sample heterogeneity were also included in the multivariate analyzes. Crude estimates of hazard ratios (HR) were presented with their respective 95% confidence interval (95% CI), regarding the association between the covariates of interest and the individual presenting some level of frailty and presence of depressive symptoms. The Wald test was performed to calculate the p-value for each coefficient of the covariates of the survival models. The Schoenfeld residual analysis statistical test was used to assess the assumption of proportional failure rates in the final Cox model. All hypothesis tests were performed with 5% significance.

### Ethical aspects

The ethical principles of voluntary and consented participation were observed through the signing of the Informed Consent form (IC) in accordance with recommendations contained in Resolution No. 466 of the National Health Council of 12 December, 2012, prior to the start of data collection. Participants were given information about the possibility of refusing to answer any questions, or even ending their participation by requesting a copy of the signed IC without this interfering in any way with their care at the Basic Health Unit. The study project was approved by the Research Ethics Committee of the Health Sciences Sector of the Universidade Federal do Paraná under opinion 4.766.196 of June 2021, and by the Ethics Committee of the Municipal Health Department under opinion 4.856.197 of July 2021.

## Results

Table 1 shows that 85 older adults completed the average follow-up time of 763 ± 88 days (620-942). There was a predominance of females (60%), age range 70-79 years (55.3%), average education level (4-8 incomplete years of study) (41.2%), self-declared white skin color (71.8%), married (52.9%) and an income of up to two minimum wages (56.5%). The participants who did not remain in the follow-up were those in the youngest age range (60-69 years; 64.5%) and female (59.7%).

At the end of the follow-up, 97.6% of older adults reported health problems with a predominance of cardiovascular diseases (70.6%). Multimorbidities reached 80% of the sample with even higher percentages of medication use (92.9%). The variable polypharmacy was observed in 43.5% of the people in the second wave.

**Table 1:** Distribution of sociodemographic characteristics (*n*=147)

VARIABLE	First wave of data collection		Second wave of data collection		Loss to follow-up	
	**(*n*=85)**	% (95%CI*)	**(*n*=85)**	% (95%CI*)	**(*n*=62)**	% (95%CI*)
Sex						
Female	51	60 (49.4; 69.8)	51	60 (49.4; 69.8)	37	59.7 (47.3; 71.0)
Male	34	40 (30.2; 50.6)	34	40 (30.2; 50.6)	25	40.3 (29.0; 52.7)
Age group						
60 to 69 years	39	45.9 (35.7; 56.4)	31	36,.5 (27.0; 47.1)	40	64.5 (52.1; 75.3)
70 to 79 years	42	49.4 (39.0; 59.8)	47	55.3 (44.7; 65.4)	18	29 (19.2; 41.3)
80 years or older	4	4.7 (1.8; 11.5)	7	8.2 (4.0; 16.0)	4	6.5 (2.5; 15.4)
Level of schooling						
Illiterate (<1 year)	4	4.7 (1.8; 11.5)	3	3.5 (1.2; 9.9)	3	4.8 (1.7; 13.3)
Low level (1-4 incomplete years)	24	28.2 (19.8; 38.6)	14	16.5 (10.1; 25.8)	16	25.8 (16.6; 37.9)
Medium level (4-8 incomplete years)	21	24.7 (16.8; 34.8)	35	41.2 (31.3; 51.8)	15	24.2 (15.2; 36.2)
High level (8 years or more)	36	42.4 (32.4; 53.0)	33	38.8 (29.2; 49.5)	28	45.2 (33.4; 57.5)
Skin color (self-declared)						
White	62	72.9 (62.7; 81.2)	61	71.8 (61.4; 80.2)	48	77.4 (65.6; 86.0)
Brown, black, yellow or indigenous	23	27.1 (18.8; 37.3)	24	28.2 (19.8; 38.6)	14	22.6 (14.0; 34.4)
Marital status						
Single	6	7.1 (3.3; 14.6)	5	5.9 (2.5; 13.0)	4	6.5 (2.5; 15.4)
Married	48	56.5 (45.9; 66.5)	45	52.9 (42.4; 63.2)	30	48.4 (36.4; 60.6)
Stable union	2	2.4 (0.6; 8.2)	4	4.7 (1.8; 11.5)	2	3.2 (0.9; 11.0)
Separated	8	9.4 (4.8; 17.5)	8	9.4 (4.8; 17.5)	5	8.1 (3.5; 17.5)
Divorced	6	7.1 (3.3; 14.6)	6	7.1(3.3; 14.6)	8	12.9 (6.7; 23.4)
Widowed	15	17.6 (11.0; 27.1)	17	20 (12.9; 29.7)	13	21 (12.7; 32.6)
Employment status						
Working	10	11.8 (6.5; 20.3)	6	7.1 (3.3; 14.6)	7	11.3 (5.6; 21.5)
Retired	55	64.7 (54.1; 74.0)	52	61.2 (50.5; 70.8)	34	54.8 (42.5; 66.6)
Retired + working	10	11.8 (6.5; 20.3)	18	21.2 (13.8; 31.0)	5	8.1 (3.5; 17.5)
Pensioner	4	4.7 (1.8; 11.5)	5	5.9 (2.5; 13.0)	8	12.9 (6.7; 23.4)
Retired + pensioner	1	1.2 (0.2; 6.4)	3	3.5 (1.2; 9.9)	1	1.6 (0.3; 8.6)
Unemployed	5	5.9 (2.5; 13.0)	1	1.2 (0.2; 6.4)	7	11.3 (5.6; 21.5)
Older adult income						
Up to 2 MW**	45	52.9 (42.4; 63.2)	48	56.5 (45.9; 66.5)	39	62.9 (50.5; 73.8)
From 2 to 4 MW**	25	29.4 (20.8; 39.8)	25	29.4 (20.8; 39.8)	14	22.6 (14.0; 34.4)
From 4 to 10 MW**	13	15.3 (9.2; 24.4)	9	10.6 (5.7; 18.9)	4	6.5 (2.5; 15.4)
> 10 MW**	0	0 (0.0; 4.3)	1	1.2 (0.2; 6.4)	1	1.6 (0.3; 8.6)
No income	2	2.4 (0.6; 8.2)	2	2.4 (0.6; 8.2)	4	6.5 (2.5; 15.4)

NOTE: *95%CI - 95% confidence interval; **MW - Brazilian minimum wage (R$1,518.00; Decree No. 12.342/2024)

Most older adults showed a low degree of adherence to social distancing and social isolation (69.4%). In the second wave, 14.1% developed depressive symptoms. Of the 85 non-frail individuals in the first wave, 51.8% evolved to pre-frailty, 2.3% to frailty, and 45.9% remained non-frail throughout the follow-up. Pre-frail and frail individuals showed a lower degree of adherence to social distancing and social isolation (n=30; 65.2%) than non-frail individuals (n=29; 74.4%). There was no association between the degree of adherence to social distancing and physical frailty (p=0.5).

**Table 2:** Association between the degree of adherence to social distancing and social isolation of the cohorts and the presence of depressive symptoms and physical frailty

Adherence to social distancing and social isolation	Physical frailty				***p*-value** ^†^
	Non-frail		Pre-frail or frail		
	**(*n*=39)**	% (95%CI*)	**(*n*=46)**	% (95%CI*)	
High	10	25.6 (14.6; 41.1)	16	34.8 (22.7; 49.2)	0.5
Low	29	74.4 (58.9; 85.4)	30	65.2 (50.8; 77.3)	
Adherence to social distancing and social isolation	Depressive symptoms				***p*-value** ^†^
	Yes		No		
	**(*n*=12)**	% (95%CI*)	**(*n*=73)**		% (95%CI*)		
High	3	25 (8.9-53.2)	23		31.5 (22.0-42.9)		0.748
Low	9	75 (46.8-91.1)	50		68.5 (57.1-78.0)	

Note: *95%CI - 95% Confidence Interval; ^†^Chi-square test, p-value <0.05

Regarding the diagnosis of Covid-19, the same occurred in 2.6% (95%CI 0.5-13.2) of the non-frail and in 8.7% (95%CI 3.4-20.3) of pre-frail and frail, p=0.369. Table 3 shows that single, separated, divorced or widowed individuals have a 62% (HR=0.38; 95%CI 0.15-0.96) lower risk of being classified as frail and 57% (HR=0.43; 95%CI 0.21-0.9) lower risk of presenting depressive symptoms compared to married individuals. Older adults who are working or pensioners have a 72% (HR=0.28; 95%CI 0.12-0.69) lower risk of being classified as frail and 60% (HR=0.40; 95%CI 0.21-0.77) lower risk of presenting depressive symptoms compared to those who are not working.

**Table 3:** Proportional hazards models for the association of the presence of frailty and depressive symptoms with sociodemographic characteristics

	Physical frailty		Depressive symptoms		
Variables	Crude HR^*^ (95%CI^†^)	Model	Crude HR^*^ (95%CI^†^)	Model
		Adjusted HR^*^ (95%CI^†^)		Adjusted HR^*^ (95%CI^†^)
Sex				
Male	1. (Ref.)	1. (Ref.)	1. (Ref.)	1. (Ref.)
Female	1.33 (0.64; 2.78)	2.55 (0.89; 7.36)	0.64 (0.4; 1.03)	1 (0.48; 2.11)
Level of Schooling				
Low	1. (Ref.)	1. (Ref.)	1. (Ref.)	1. (Ref.)
Medium	1.41 (0.55; 3.59)	1.78 (0.6; 5.29)	1.68 (0.86; 3.27)	1.32 (0.63; 2.76)
High	1.89 (0.78; 4.56)	2.25 (0.74; 6.85)	1.68 (0.87; 3.25)	1.44 (0.65; 3.17)
Age range				
60 to 69 years	1. (Ref.)	1. (Ref.)	1. (Ref.)	1. (Ref.)
70 years or older	0.72 (0.38; 1.36)	0.78 (0.33; 1.81)	0.87 (0.54; 1.4)	0.85 (0.44; 1.65)
Race/color				
White	1. (Ref.)	1. (Ref.)	1. (Ref.)	1. (Ref.)
Non-white	0.87 (0.39; 1.91)	0.93 (0.38; 2.29)	1.24 (0.72; 2.12)	1.67 (0.89; 3.16)
Marital status				
Married/Stable union	1. (Ref.)	1. (Ref.)	1. (Ref.)	1. (Ref.)
Single/Divorced/Widowed	0.75 (0.39; 1.43)	0.38 (0.15; 0.96)^§^	0.54 (0.33; 0.88)	0.43 (0.21; 0.9) ^§^
Multimorbidity				
Yes	1. (Ref.)	1. (Ref.)	1. (Ref.)	1. (Ref.)
No	0.66 (0.29; 1.51)	1.27 (0.43; 3.78)	0.69 (0.37; 1.26)	1.14 (0.54; 2.41)
Employment status				
Not working	1. (Ref.)	1. (Ref.)	1. (Ref.)	1. (Ref.)
Working/Pensioner	0.52 (0.26; 1.03)	0.28 (0.12; 0.69)^§^	0.64 (0.39; 1.05)	0.40 (0.21; 0.77)^§^

Note: ^*^HR - hazard ratio; ^†^95%CI = 95% Confidence Interval; Wald test^§^ 0.01 ≤p-value < 0.05.

## Discussion

When assessing the effects of social distancing and social isolation in the cohorts from the first to the second wave of data collection, more than half of the older adults (51.8%) evolved to pre-frailty, 2.3% to frailty, and 45.9% remained non-frail. The transition from frailty can occur in a short or long period of time; the magnitude of this period has not yet been established, since the pathophysiology of physical frailty is quite complex. The high percentage of pre-frailty in the population of older adults is observed in studies developed in different contexts, both internationally and nationally.^16^^-^^18^ A retrospective cohort study analyzed data from the longitudinal Survey of Health, Ageing and Retirement in Europe (SHARE) carried out in 28 countries (age 65 years and older). At baseline, 8,133 were non-frail. Of this sample, 34.4% (n=2,798) developed pre-frailty and 3.0% (n=247) developed frailty within two years of baseline.^19^

When comparing the percentages of physical frailty with those observed in the studies, although the contexts in which the studies were conducted varied greatly, the pre-frailty condition predominated in most studies. In view of this, there is growing interest in interventions in pre-frailty, since this stage is sensitive to changes and can be clinically reversible,^20^ which highlights the importance of its early identification and interventions to reverse it or prevent its progression. Low adherence to social distancing and social isolation was observed in approximately 75% of non-frail older adults, which is a higher percentage than that of non-frail with high adherence (25.6%, 14.6-41.1). Low adherence was also observed in pre-frail and frail subjects (65.2%). The restrictive measures adopted during the Covid-19 pandemic also contributed to the transition to levels of physical frailty, as more than 50% transitioned to pre-frailty and 2.3% to frailty. A prospective longitudinal study of 119 older adults from the Montreal community (Canada) (≥ 70 years) showed that home confinement was associated with increased physical frailty (p≤0.038).^20^

In the present study, 14.1% of the sample developed depressive symptoms. A similar result was observed in the analysis of the second wave of a cohort study with 7,609 Medicare beneficiaries in the United States of America, which showed that 15% of older adults presented depressive symptoms.^21^ An even more expressive percentage was found in a systematic review with meta-analysis that aimed to assess the prevalence of depressive symptoms in mainland China. Eighty-one eligible studies were analyzed and a combined global prevalence of depressive symptoms of 20.0% (95% CI; 17.5-22.8) was identified.^22^ Depression in older adults is worrying, as this is a disorder with declining energy and the potential to affect all aspects of life, in addition to aggravating and/or triggering other conditions, such as physical frailty.^23^ Furthermore, in old age, as a rule, the person faces unwanted changes, losses and feelings of grief, which makes treatment more difficult and time-consuming.

The adoption of restrictive measures may have contributed to an increase in the percentages of depressive symptoms in older adults, even though a low incidence was observed in the present study in relation to incidence in the country. In a meta-analysis conducted in Minas Gerais (Brazil), a prevalence of 21.0% (95% CI; 18.0-25.0; I2=98.3%) of depressive symptoms was found in community-dwelling older adults with a variation of 7.10% to 39.6%, respectively, for a study conducted in the South and Northeast regions of the country. Thus, the South region, where the present study was conducted, had the lowest percentage.^10^ Note that the sample of the present study is composed of older adults with a high level of education, retirees from the South region of Brazil, which is one with the best socioeconomic indicators. Therefore, the fact that this sample has more favorable sociodemographic conditions may influence higher rates of coping, resilience, and social support, resulting in lower rates of depression.

Age may also be a protective factor for mental health in the context of the pandemic, since retired older adults with a high level of education would not have to deal with changes in their work routine, fear of unemployment, financial insecurity and poverty, which are serious economic consequences that seriously affect the mental health of people working in the job market.^24^ Regarding the degree of adherence to social distancing and social isolation in the present study, it was significantly low (69.4%), only 30.6% achieved a high degree of adherence to restrictive measures. The low degree of adherence to social distancing and social isolation by older adults has been observed in several studies, with great variability in percentages. This is justified because the definition of social isolation or social distancing varies between studies and different epidemic periods, making comparisons difficult, both between countries and between groups or individuals within the same country.^25^

National studies have identified a low degree of adherence to social distancing and social isolation among older adults, particularly among participants in the second wave of the ELSI-Brazil from 70 municipalities located in the five major regions of the country, which examined the prevalence of social distancing in a sample of 6,149 subjects (mean age 63.4 years). Social distancing was defined as not having left home in the previous seven days. Only 32.8% of the study participants did not leave home during the period considered, 36.3% left home between one and two times, 15.2% between three and five times and 15.7% went out every day.^25^ A cross-sectional study of 4,035 participants from the second wave of the ELSI-Brazil identified that 37.2% of them went out for essential activities, and living in the South Region increased the chance of older adults going out for essential activities (OR 1.77; 95% CI: 1.01-3.1), as the distancing measures were more flexible, since the incidence of Covid-19 was lower at the time of the interviews compared to other regions in the country.^26^

The need to work in order to contribute to the family income may be linked to the low adherence of older adults to restrictive measures. A study of 9,173 Brazilians investigated the adherence to social distancing. Retired older adults or those who were no longer working before the pandemic showed greater adherence to social distancing measures (OR 40.4%; 95%CI 34.8-46.3 and OR 41.7%; 95%CI 33.8-50.0), while approximately 10% of older adults who continued working from home still did not adhere to social distancing (95%CI 5.4-17.4). Of those who performed some essential activity during the pandemic, 44.2% (95%CI: 33.9-55.1) did not adhere to social distancing.^27^ Of the 14.1% of older adults who developed depressive symptoms, 75% showed low adherence to social distancing and social isolation. This datum is corroborated by a study of 1,005 older adults (≥65 years) conducted in Germany with the aim to identify mental well-being during the restrictive measures of Covid-19. The findings did not indicate a worsening in mental well-being and showed similar or even lower numbers compared to studies prior to the pandemic.^28^

Although an association between depressive symptoms and social distancing and social isolation has not been found in the present investigation, the magnitude of the emotional consequences of social distancing during Covid-19 should be considered. A recent study showed that reduced social connectivity during the Covid-19 pandemic increased the risk of presenting symptoms of depression by 17.24 times (95% CI; 13.20-22.50).^29^

In a study of 477 older adults (mean age of 71.6 years), it was found that greater social isolation predicted greater depression (p<0.001).^30^ For older adults confined to their homes, doing things alone is often unattainable due to limited physical and mental capacity, which can cause common symptoms of depression, such as decreased feelings of self-control and competence^31^. The initial characteristics of the sample, with non-frail older adults, may have led to the lack of association between social distancing and social isolation and depression.

In addition to exercising their care skills during the pandemic, nursing professionals must create bonds with the population of older adults in the community to establish a relationship of trust and thus maximize their service strategies. In this regard, nurses need to pay attention to effective, clear communication that is adapted to the reality experienced by the population.^32^

Single, separated, divorced or widowed older adults have a 62% (HR=0.38; 95%CI 0.15-0.96) lower risk of being classified as frail and a 57% (HR=0.43; 95%CI 0.21-0.9) lower risk of presenting depressive symptoms compared to married individuals. Converging data were observed in the China Health and Retirement Longitudinal Study (CHARLS) conducted with 14,351 older adults (≥ 60 years). The aim of the study was to investigate the incidence of frailty in the population of Chinese older adults living in the community and explore the risk factors and protective factors for frailty. It was observed that the marital status (single, divorced and widowed) (OR 0.43; 95% CI; 0.27-0.67) was a protective factor against physical frailty.^17^

Regarding the significant association between depressive symptoms and the marital status variable, married subjects or those in a stable union develop depressive symptoms more quickly and in a shorter observation time. These findings are related to different factors, among which the greater exposure to the risk of suffering depletion processes resulting from marital relationships. In view of this, the marital status may be related to the development of depressive symptoms in older adults. Health professionals should pay attention to older adults at risk of developing depressive symptoms, especially those who are married or in a stable union, and seek to offer social support to prevent this problem.

Older adults who work or are pensioners are at a 72% lower risk of being classified as frail and a 60% lower risk of presenting depressive symptoms compared to those who are not working. Older adults out of the job market, with reduced income and problems related to financial autonomy, experience a change in their role, from provider to financially dependent, which can favor the emergence of depressive symptoms.^33^ These data are an alert to the need for a closer look at characteristics of the sociodemographic profile that are often neglected when it comes to health care of older adults.

The diagnosis of Covid-19, during the follow-up of the cohorts did not differ according to the frailty condition. These findings are corroborated by data from a prospective cohort with 4,510 participants. The condition of pre-frailty (OR 0.9; 95% CI; 0.8-1.1) and frailty (OR 0.9; 95% CI; 0.7-1.2) was not associated with Covid-19 diagnosis.^34^ A divergent finding was found in a study conducted in England with 383,845 participants (aged between 37 and 73 years) from the UK Biobank. A higher risk of Covid-19 was observed in pre-frail individuals (RR 1.47; 95% CI; 1.26-1.71) and frail individuals (RR 2.66; 95% CI; 2.04-3.47).^35^

The limitations of the study are: sample losses, lack of standardization of the different instruments and cutoff points used in the studies to evaluate the main variables, making comparisons difficult; the use of a scale without testing and validation to assess the degree of adherence to social distancing and social isolation, in addition to memory bias in relation to the time evaluated; the self-report questions of some instruments may have been impaired by memory failures, thereby contributing to less accurate information.

The importance of health professionals, especially nurses, in continuously monitoring the physical frailty of older adults and establishing an action plan aimed at reversing the status of frail or pre-frail individuals to a non-frail condition is highlighted. To this end, it is essential to encourage the adoption of practices that are already established in the literature, which are pillars for preventing the progression of physical frailty, including: appropriate caloric and protein support, vitamin D supplementation, reduction of polypharmacy, and the practice of resistance and aerobic physical exercises. Actions that involve encouraging the continuous practice of physical activities and exercises are essential.

Nursing professionals play a major role in health services, especially in primary health care. As such, they are the main characters, creators of guiding strategies for the implementation and dissemination of measures aimed at preventing depressive symptoms and physical frailty in older adults.

The conclusion of this study is that more than half of the sample evolved to a pre-frail condition. The low incidence of depressive symptoms was attributed to the resilience commonly acquired by older adults over the course of their lives. Most older adults showed a low degree of adherence to social distancing and social isolation measures during the Covid-19 pandemic. Social distancing and social isolation did not generate depressive symptoms and did not change the condition of physical frailty in older adults in primary health care. Single, separated, divorced or widowed subjects had a lower risk of developing depressive symptoms and being classified as pre-frail and frail compared to married subjects. Older adults who are working or are pensioners are at a lower risk of developing depressive symptoms and being classified as pre-frail and frail.

This study supports strategies for gerontological practice by directing the monitoring of older adults at risk in order to prevent the transition to a condition of frailty and depressive symptoms in primary health care. Among the strategies, it is essential to encourage the return to the practice of activities that were interrupted during the pandemic, and monitor older adults with a high family income, who do not work and are married or in a stable union for the risk of depressive symptoms and physical frailty.
